# STmiR: A Novel XGBoost-based framework for spatially resolved miRNA activity prediction in cancer transcriptomics

**DOI:** 10.1371/journal.pone.0322082

**Published:** 2025-09-09

**Authors:** Jiaqi Yuan, Peng Xu, Zheng Ye, Wenbin Liu

**Affiliations:** 1 Institute of Computational Science and Technology, Guangzhou University, Guangzhou, China; 2 School of Computer Science of Information Technology, Qiannan Normal University for Nationalities, Duyun, Guizhou, China

## Abstract

MicroRNAs (miRNAs) are critical regulators of gene expression in cancer biology, yet their spatial dynamics within tumor microenvironments (TMEs) remain underexplored due to technical limitations in current spatial transcriptomics (ST) technologies. To address this gap, we present STmiR, a novel XGBoost-based framework for spatially resolved miRNA activity prediction. STmiR integrates bulk RNA-seq data (TCGA and CCLE) with spatial transcriptomics profiles to model nonlinear miRNA-mRNA interactions, achieving high predictive accuracy (Spearman’s ρ > 0.8) across four major cancer types (breast, lung, ovarian, prostate), with performance further confirmed through direct comparison with experimentally measured miRNA expression in an independent spatial transcriptomics dataset. Applied to 10X Visium ST datasets from nine cancers, STmiR identifies six pan-cancer conserved miRNAs (e.g., hsa-miR-21, hsa-let-7a) consistently ranked in the top 40 across malignancies, and uncovers cell-type-specific regulatory networks in fibroblasts, B cells, and malignant cells. A breast cancer case study demonstrates STmiR’s utility in uncovering biologically relevant miRNA-target relationships and their association with key cancer pathways. By enabling spatial mapping of miRNA activity, STmiR provides a transformative tool to dissect miRNA-mediated regulatory mechanisms in cancer progression and TME remodeling, with implications for biomarker discovery and precision oncology.

## Introduction

MicroRNAs (miRNAs) are small, non-coding RNA molecules that play critical roles in post-transcriptional gene regulation by binding to target messenger RNAs (mRNAs), leading to their degradation or translational repression [1,2]. These regulatory mechanisms are essential for controlling a wide range of biological processes, including cell differentiation, development, and disease progression [3,4]. Dysregulation of miRNA expression has been implicated in various diseases such as cancer, cardiovascular disorders, and neurodegenerative conditions, highlighting the importance of miRNAs as both biomarkers and potential therapeutic targets [5,6]. As such, accurate profiling of miRNA expression is crucial for uncovering the underlying regulatory networks and understanding gene regulation in both health and disease [7].

However, measuring miRNA expression, especially in the spatial context of tissues, remains a significant technical challenge [8]. Spatial transcriptomics (ST) technologies, while offering the ability to spatially resolve mRNA expression patterns within tissues, are not optimized for capturing miRNAs due to their small size and distinct structural properties [9]. This limitation has hindered the study of miRNA expression profiles in spatial transcriptomics, leaving gaps in our understanding of the spatial organization of gene regulation by miRNAs.

Several computational approaches have been developed to address this challenge by inferring miRNA activity indirectly. Current computational approaches (e.g., GenMIR++ and MAGIA) rely heavily on linear correlation models to infer miRNA-mRNA interactions. These methods assume a simplistic inverse relationship between miRNA and target mRNA expression levels, neglecting the nonlinear dynamics inherent in biological systems. For instance, GenMIR++ fails to account for cooperative miRNA binding or context-dependent regulatory effects, leading to reduced accuracy in complex tissues such as tumors [10]. To improve on these limitations, more recent machine learning-based approaches have emerged. For example, miRLAB integrates various biological features to predict miRNA-mRNA interactions [11], and DeepMirTar applies deep learning to enhance miRNA target prediction and infer miRNA activity [12]. Additionally, single-cell RNA sequencing (scRNA-seq) has spurred the development of methods such as miRSCAPE, which leverages the reduction in target mRNA expression to infer miRNA activity in single cells [13]. Although miRSCAPE is capable of predicting miRNA activity from single-cell sequencing data, the unique nature of spatial transcriptomics, where each spot resembles bulk data composed of multiple cells, makes it more appropriate to utilize models trained on bulk RNA-seq data for miRNA activity inference. The advancement of computational methods in spatial transcriptomics also provides new avenues for inferring molecular features that are not directly measured, highlighting the potential for model-based predictions [14,15].

Here, we present STmiR, the first machine learning framework that integrates XGBoost with spatial transcriptomics to predict miRNA activity. The complete workflow of our approach is outlined in S1 Fig. The process begins by training a predictive model on large-scale, paired miRNA-mRNA bulk RNA-seq datasets from sources like TCGA and CCLE. This trained model is then applied to the mRNA expression profiles from spatial transcriptomics data to infer miRNA activity for each spatial spot. Unlike single-cell tools such as miRSCAPE, which are optimized for single-cell resolution but struggle with spatially aggregated ‘spot-level’ data, STmiR leverages these bulk RNA-seq-trained models to capture nonlinear miRNA-mRNA relationships across heterogeneous tissue regions. This approach enables accurate spatial mapping of miRNA activity while preserving the multicellular complexity of tumor microenvironments, facilitating downstream functional analyses such as building cell-type-specific regulatory networks.

By training a model on matched miRNA-mRNA tissue samples, STmiR can learn the underlying relationships between RNA and miRNA. The trained model is then applied to spatial transcriptomics data, allowing the prediction of miRNA activity for each spot, thereby generating spatial maps of miRNA activity. This approach provides a powerful new tool for studying miRNA dynamics in spatial contexts, offering insights into the spatial regulation of gene expression that were previously inaccessible. In parallel, we conducted a pan-cancer analysis to identify highly expressed common and specific genes across cell types in different cancer types, providing further insights into the molecular underpinnings of cancer biology.

Throughout this manuscript, we make a clear distinction between “miRNA expression/abundance,” which refers to experimentally measured values (e.g., from miRNA-seq), and “predicted miRNA activity.” We define **“predicted miRNA activity”** as the quantitative estimation of a miRNA’s regulatory function and strength, as inferred by the STmiR model from the local mRNA expression profile. It serves as a composite metric reflecting the miRNA’s regulatory impact on its target gene network.

In summary, STmiR addresses the limitations of current miRNA profiling methods in spatial transcriptomics by leveraging machine learning to accurately infer miRNA expression activity from mRNA profiles. By enabling spatially resolved miRNA activity mapping, STmiR offers a powerful tool for investigating the spatiotemporal dynamics of miRNA-mediated regulation in tumor microenvironments, with potential applications in identifying therapeutic targets and deciphering mechanisms of cancer progression.

## Methods

### Data Source

#### Bulk-RNAseq.

We procured the matched bulk RNA-Seq and miRNA-Seq gene expression data for cell lines from the Cancer Cell Line Encyclopedia [16] (CCLE, as described by Barretina et al. in 2012) and Pan-cancer data from The Cancer Genome Atlas (TCGA) [17] via the URLs provided at https://pancanatlas.xenahubs.net. The batch effects normalized mRNA-seq and miRNA-seq data were obtained from GDC(https://portal.gdc.cancer.gov/). Notably, the miRNA sequencing data encompassed 744 miRNAs across 10,818 samples, while the mRNA sequencing data comprised 20,532 genes across 11,060 samples. We utilized the TPM (Transcripts Per Kilobase Million) normalized RNA-seq data from CCLE [18] (as reported by Ghandi et al. in 2019).

Initially, following outlier processing and logarithmic transformation of the original TCGA and CCLE data, we selected the matched mRNA and miRNA samples from nine distinct sites. Subsequently, considering the miRNA names, we removed the long suffixes (such as -3p), eliminated duplicates of the same miRNA name, and replaced them with the median value. Z-score normalization was then employed for data standardization. Finally, we utilized rank-based methods to integrate the sequencing data from both TCGA and CCLE sources, with the aim of eliminating batch effects.

### Integration and normalization of TCGA and CCLE data

To eliminate batch effects between the TCGA and CCLE datasets, we implemented a rigorous rank-based normalization and integration pipeline. The detailed steps are as follows:

Step 1: Independent Pre-processing of Datasets

1. Data Filtering: We first processed the mRNA and miRNA expression matrices from TCGA and CCLE independently. We retained only the genes and miRNAs that were common to both datasets. Genes/miRNAs with zero expression in more than 90% of samples were removed.
2. Log Transformation: To stabilize variance and reduce the influence of outliers, we applied a log2(x + 1) transformation to the expression values (e.g., TPM or FPKM).

Step 2: Rank-Based Normalization

1. Intra-Sample Ranking: Within each sample (i.e., each column of the TCGA and CCLE matrices), we ranked the log2-transformed expression values of all genes from lowest to highest and calculated their percentile rank. This step converts the expression distribution of each sample into a uniform distribution from 0 to 1, removing sample-specific distributional differences.
2. Cross-Sample Rank-based Inverse Normal Transformation: For each gene (i.e., each row of the matrices), we collected its percentile ranks across all samples. These ranks were then mapped to a standard normal distribution. Specifically, each rank value was replaced by the Z-score from a standard normal distribution corresponding to its quantile. This transformation ensures that each gene’s expression values follow a standard normal distribution across all samples, making the TCGA and CCLE data distributionally comparable and effectively removing technical variations arising from different platforms, labs, or batches.

Step 3: Data Integration and Final Scaling

1. Data Concatenation: After the normalization process, the TCGA and CCLE data matrices were directly concatenated by samples (columns) to form a single, unified expression matrix.
2. Final Scaling of Features and Targets: Before feeding the data into the XGBoost model, we applied Min-Max Scaling to both the mRNA expression data (features) and the miRNA expression data (targets), scaling their values to a [0, 1] range. This step helps to optimize the training efficiency and performance of the machine learning model.

### scRNA-seq

We conducted an extensive analysis of nine single-cell RNA sequencing (scRNA-seq) expression profiles obtained from the TISCH2 [19] database (http://tisch.comp-genomics.org/gallery/), which provides comprehensive resources including differential gene expression, cell-type annotations, and associated metadata. These datasets represent diverse human cancer types, offering a broad scope for our investigation into tumor heterogeneity and microenvironment interactions.

The datasets analyzed include:

- **BRCA_EMTAB8107** (33,043 cells from 14 breast cancer patients, 10x Genomics), which comprises a substantial cohort of primary breast cancer samples.
- **CESC_GSE168652** (22,998 cells from 1 cervical cancer patient, 10x Genomics), providing a focused profile of a single primary cervical cancer sample.
- **CRC_GSE166555** (66,050 cells from 12 colorectal cancer patients, 10x Genomics), offering a large-scale dataset from primary colorectal tumors.
- **Glioma_GSE103224** (17,185 cells from 8 glioma patients, Microwell), which includes primary glioma samples sequenced using the Microwell platform.
- **NSCLC_EMTAB6149** (40,218 cells from 5 non-small cell lung cancer patients, 10x Genomics), representing primary non-small cell lung cancer samples.
- **SKCM_GSE72056** (4,645 cells from 19 metastatic melanoma patients, Smart-seq2), which provides insights into metastatic melanoma using Smart-seq2 sequencing technology.
- **OV_EMTAB8107** (24,781 cells from 5 ovarian cancer patients, 10x Genomics), capturing the cellular diversity of primary ovarian cancer samples.
- **PRAD_GSE137829** (8,640 cells from 6 prostate cancer patients, 10x Genomics), focused on primary prostate cancer tumors.
- **CRC_EMTAB8107** (23,176 cells from 7 colorectal cancer patients, 10x Genomics), offering another dataset of primary colorectal cancer samples.

To facilitate downstream analysis, scRNA-seq data were first converted from H5 to Loom format using the Seurat4.3 [20] and loomR packages. This conversion enabled efficient handling of large-scale single-cell datasets, which were subsequently subjected to comprehensive bioinformatic analysis and processing. Standardized protocols appropriate to each sequencing platform were applied to ensure the accuracy and reproducibility of our results across these diverse cancer types. This dataset collection and processing pipeline provided a robust foundation for our exploration of cancer biology at single-cell resolution.

### Spatial transcriptomics

We obtained 10X Visium Spatial Transcriptomics (ST) data from 10x Genomics (https://www.10xgenomics.com/datasets), selecting datasets for breast cancer, non-small cell lung cancer, ovarian cancer, melanoma, cervical cancer, intestine cancer, glioma, colon cancer, and prostate cancer. For each cancer type, we downloaded both the spatial imaging data and the corresponding filtered feature matrix files. The gene-spot matrices generated from these ST and Visium samples were processed and analyzed using the scanpy package [21]. In the initial steps, we applied basic filtering to the spots based on total counts and the number of expressed genes.

To ensure high data quality, we systematically identified and removed outliers from the spatial transcriptomic datasets. Specifically, in the breast cancer dataset, we excluded five cells with expression counts exceeding 38,000, as they represented extreme outliers. Additionally, genes expressed in fewer than ten cells were filtered out, ensuring that the analysis focused only on the most robustly and consistently expressed genes.

Following these preprocessing steps, we normalized the Visium count data using the standardization method implemented in scanpy and performed a log10 transformation. This transformation compressed the dynamic range of the data, improving both the stability and sensitivity of downstream analyses. To prioritize the most informative features, we identified highly variable genes and selected the top 2,000 feature genes. These genes exhibited significant expression differences across cells, making them critical for subsequent cell type identification and analysis of biological processes.

Finally, the expression data were scaled, and we employed Uniform Manifold Approximation and Projection (UMAP) for dimensionality reduction based on gene expression profiles. Clustering was then performed, allowing us to investigate cellular heterogeneity and spatial patterns within the cancer samples, providing key insights into tumor microenvironment organization and cell-type diversity.

### Model construction and performance evaluation

A schematic of the model construction and evaluation pipeline is provided in S2 Fig. In this study, we selected Extreme Gradient Boosting (XGBoost) as the primary model due to its robust performance in handling sparse data, reducing overfitting, and offering faster training times compared to other algorithms. XGBoost is an ensemble learning method based on decision trees, where a series of trees are iteratively constructed to improve predictions. Each successive tree is trained to minimize the residual errors from the previous model by optimizing a loss function. [22].

We developed a predictive model leveraging the XGBoost implementation in Python, trained on the integrated bulk RNA-seq datasets containing paired mRNA and miRNA expression profiles from TCGA and CCLE. In this framework, the rank-normalized mRNA expression levels were utilized as input features, while the corresponding miRNA expression levels served as the target output variables for training. To maximize the model’s predictive performance, we performed hyperparameter tuning through an exhaustive Grid Search, systematically evaluating combinations of parameters (such as learning rate, tree depth, and number of estimators) to identify the optimal configuration [23].

The dataset used for model development was compiled from the TCGA and CCLE repositories, with analysis focused on genes common to both datasets. To mitigate batch effects, rank-based normalization was applied prior to integration, ensuring consistent gene expression profiles across datasets. For miRNA profiling, a curated database was constructed by selecting only miRNAs shared between TCGA and CCLE, following guidelines to reduce false positive annotations [24] and ensure biological relevance across the cancer types analyzed. Additionally, mRNA features included in the model were intersected with spatial transcriptomics data to enhance biological interpretability.

Preprocessing involved min-max normalization of bulk mRNA and miRNA expression data, followed by partitioning the standardized dataset into training and testing subsets. The XGBoost algorithm was implemented with default parameters and subsequently optimized using a grid search to identify the best-performing hyperparameter configuration. Model performance was evaluated by calculating the Spearman correlation coefficient between predicted miRNA activity and observed miRNA expression, quantifying the model’s predictive accuracy.

To benchmark the performance of XGBoost, we also constructed and evaluated several other regression models, including ridge regression, Lasso regression, random forest regression, and a feed-forward neural network. The performance of all models was rigorously assessed using multiple metrics. We calculated the Spearman correlation coefficient between predicted and observed miRNA expression to quantify the model’s accuracy in ranking miRNA levels. Additionally, we used Mean Squared Error (MSE), Mean Absolute Error (MAE), and the coefficient of determination (R²) to compare the predictive accuracy across all tested models. These metrics were averaged across five independent cross-validation folds to ensure the generalizability of our results and to mitigate overfitting. This comprehensive evaluation framework allowed us to demonstrate the superior performance of XGBoost for this prediction task.

### External validation with measured spatial miRNA data

To directly validate the predictive accuracy of STmiR on spatial data, we utilized an independent breast cancer spatial transcriptomics dataset from the China National GeneBank (CNGB) database (accession: STDS0000038), which contains concurrent measurements for both mRNA and miRNA. The raw dataset comprised 4,325 spots and 36,601 genes.

To enhance the signal-to-noise ratio of the input data, we first applied the DeepGFT [25] tool, a deep learning method based on graph Fourier transform for denoising spatial transcriptomics data. This procedure yielded a refined expression matrix of 4,325 spots by 12,000 genes.

For this validation exercise, the STmiR model was specifically trained on breast cancer data by integrating TCGA and CCLE datasets. First, highly variable genes from the STDS0000038 spatial data were identified. These genes were then intersected with the common genes between TCGA and CCLE, resulting in a final set of 897 gene features for model input. Common miRNAs between TCGA and CCLE were selected as prediction targets (346 miRNAs). To mitigate batch effects, the expression data from TCGA and CCLE (1,090 total samples) were rank-normalized prior to training the XGBoost model.

The trained model was then applied to the denoised spatial gene expression data to predict miRNA activity. The predicted miRNA expression levels were subsequently compared against the experimentally measured miRNA expression from the STDS0000038 dataset. The concordance for the common miRNAs was evaluated using the Pearson correlation coefficient and the corresponding p-value.

### Application of the model to spatial transcriptomics

To extend our bulk RNA-seq-trained model to spatial transcriptomics (ST) data, we first identify the overlapping genes between the bulk RNA-seq and ST datasets. After completing the training of the XGBoost model on bulk RNA-seq data, we input the spatial transcriptomics gene expression data into the pre-trained model to predict miRNA activity across spatially defined spots.

Our workflow integrates miRNA activity predictions with cell-type deconvolution results using a strategy based on dominant cell-type assignment. The steps are as follows:

- Prediction of Bulk miRNA Activity per Spot: The STmiR model was applied directly to the bulk mRNA expression profile of each spatial spot. This generated a single “predicted miRNA activity” value for each miRNA at each spot.
- Estimation of Cell-Type Composition: In parallel, we used the cell2location algorithm, with an annotated single-cell RNA-seq dataset as a reference, to deconvolve the cellular composition of each spatial spot, yielding an estimated relative abundance for each cell type within each spot.
- Attribution of miRNA Activity to Dominant Cell Types: To infer the cell-type-specific activity of a miRNA, we linked its predicted activity to the dominant cell type in a given spot.

1) First, we identified spots that were predominantly occupied by a single cell type. A spot was assigned a dominant cell type if that type’s relative abundance was the highest among all cell types within that spot (or exceeded a predefined threshold).
2) Next, for this subset of spots, we attributed the high predicted miRNA activities to their corresponding dominant cell type. By identifying miRNAs that consistently exhibited high activity across all spots dominated by a particular cell type (e.g., fibroblasts), we designated them as candidate miRNAs with high activity in that cell type.

We chose this intuitive approach as it directly links the molecular features of a spatial region to its primary cellular identity. By focusing on spots with relatively homogeneous cell populations, we can more confidently assign the miRNA activities—predicted from a mixed-cell signal—to a specific cell type.

Cell2location is capable of accounting for biological variability across different cell types, while also incorporating technical factors inherent in ST data generation, as described by Kleshchevnikov et al [26].

### Biofunctional analysis

We perform a comprehensive differential expression analysis across various malignant and non-malignant cell types, including cancer cells, fibroblasts, myofibroblasts, and B cells, for each cancer type. This analysis allows us to identify miRNAs with significantly differential predicted activity in these cell populations.

Following this, we construct a regulatory network of miRNAs and associated diseases using the **miRNet** platform [27] (https://www.mirnet.ca/), enabling us to explore the broader biological implications of these miRNAs. To further elucidate the functions of the identified miRNAs, we extract their target genes from the **HMDD** database [28], which provides curated information on miRNA-disease associations.

After identifying miRNA target genes, we conduct pathway enrichment analysis using **Metascape [29]**, which helps to uncover the biological pathways in which these target genes are involved. This step is crucial for understanding the functional roles of miRNAs within different cellular contexts.

Finally, we assess the correlation between the predicted miRNA activity and the expression levels of their target genes. A positive correlation suggests that the miRNA may act as a promoter of its target gene’s expression, whereas a negative correlation indicates that the miRNA likely suppresses or dysregulates the expression of its target gene. These insights into miRNA-target interactions provide important clues about the regulatory mechanisms underlying cancer biology.

### Statistical analysis

To ensure the accuracy and reliability of our findings, we employed a multifaceted statistical approach tailored to address the unique challenges of spatial transcriptomics data. The performance of the predictive model was rigorously evaluated using Spearman’s rank correlation coefficient, chosen for its robustness to non-normal distributions and outliers inherent in sparse spatial datasets. This non-parametric measure quantified the consistency between predicted miRNA activity ranks and experimentally observed values, with higher coefficients (e.g., ρ > 0.5) indicating strong alignment between model predictions and biological reality. Complementing this, traditional regression metrics—including mean squared error (MSE), mean absolute error (MAE), and the coefficient of determination (R²)—were calculated to assess prediction accuracy. These metrics were averaged across five independent cross-validation folds to mitigate overfitting and ensure generalizability, with lower MSE/MAE and higher R² values collectively guiding the selection of the optimal XGBoost configuration.

To dissect miRNA-target interactions, we performed Pearson correlation analysis between predicted miRNA activity levels and the expression of their experimentally validated target genes (curated from HMDD and miRNet databases). Interactions with absolute correlation coefficients exceeding 0.3 and adjusted P-values below 0.05 (Bonferroni-corrected) were deemed biologically significant. This dual threshold strategy balanced sensitivity and specificity, filtering out spurious associations while retaining mechanistically plausible links. For differential activity analysis, Wilcoxon rank-sum tests compared miRNA activity distributions between malignant and non-malignant cell populations, with Benjamini-Hochberg false discovery rate (FDR < 0.1) correction applied to account for multiple hypothesis testing.

Pathway enrichment analysis further contextualized the functional roles of differentially active miRNAs. Target genes were analyzed using Metascape, where hypergeometric testing against Gene Ontology Biological Processes and KEGG pathways identified overrepresented terms (adjusted P < 0.05). Visualization of regulatory networks was conducted in Cytoscape (v3.9.1), with edges weighted by correlation strength and nodes annotated by functional roles, while Matplotlib (v3.7.1) and Seaborn (v0.12.2) generated diagnostic plots such as violin plots for distribution comparisons and scatterplots for correlation trends. All statistical workflows, including code and preprocessing pipelines, are publicly accessible on GitHub to ensure full reproducibility, adhering to FAIR principles for open science.

This integrative strategy—combining non-parametric rank comparisons, regression diagnostics, hypothesis testing with multiplicity control, and pathway-centric functional annotation—provided a robust foundation for interpreting spatially resolved miRNA activity and its implications in cancer biology.

## Results

### STmiR accurately predicts miRNA activity and is validated on spatial data

**Fig 1:**
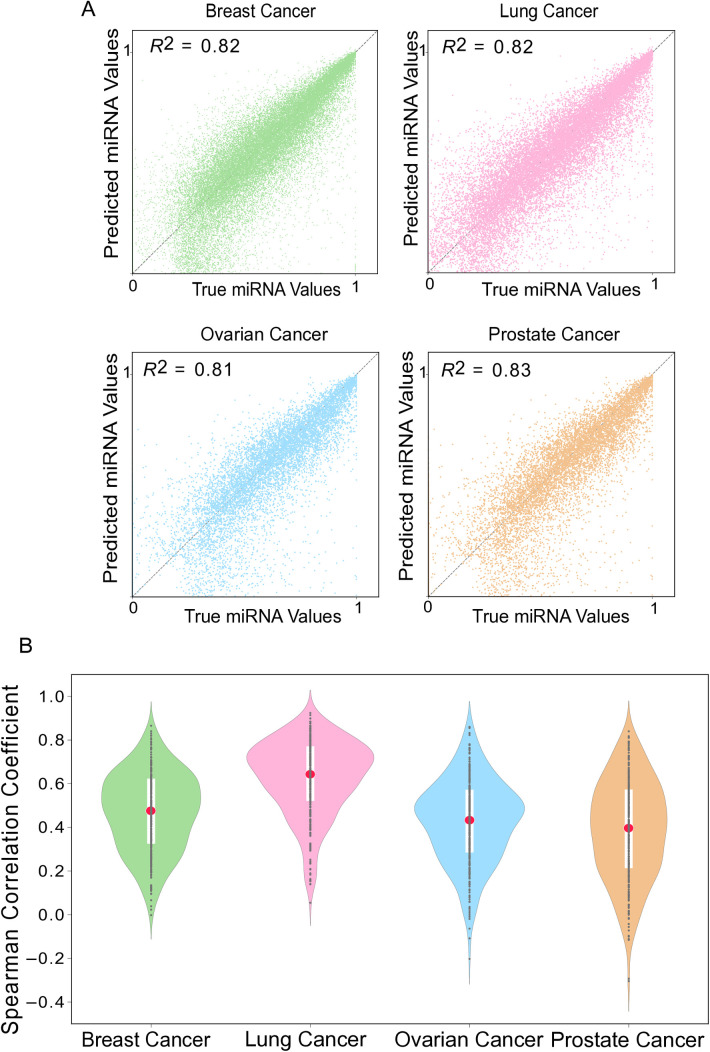
Performance of XGBoost in miRNA Activity Prediction Using Bulk RNA-seq Data. (A) Scatterplot of predicted versus observed miRNA expression levels. The plot illustrates the correlation between XGBoost-predicted miRNA activity (y-axis) and experimentally measured miRNA expression (x-axis) across four cancer types: breast cancer (BRCA), lung adenocarcinoma (LUAD), ovarian serous carcinoma (OV), and prostate adenocarcinoma (PRAD). Each point represents a miRNA-sample pair, with the solid line indicating the linear regression fit (slope = 0.89, 95% CI: 0.85–0.93). Shaded regions denote 95% confidence intervals. High concordance (Spearman’s ρ > 0.8) underscores the model’s accuracy in capturing miRNA expression trends. (B) Distribution of Spearman correlation coefficients across cancer types. Violin plots summarize the pairwise Spearman correlations between predicted and true miRNA expression levels for each cancer type. Individual points represent specific miRNAs, while the central red line marks the median correlation (BRCA: ρ = 0.82; LUAD: ρ = 0.79; OV: ρ = 0.76; PRAD: ρ = 0.84). The width of each violin reflects the density distribution, highlighting tighter clustering in BRCA and PRAD compared to LUAD. Coefficients closer to 1 indicate stronger rank consistency, demonstrating XGBoost’s robustness in modeling miRNA activity across heterogeneous cancer datasets.

To construct a robust predictive model, we first evaluated a range of machine learning regression techniques on bulk RNA-seq data from TCGA and CCLE across four major cancer types: breast, lung, ovarian, and prostate. Among the tested models, XGBoost exhibited superior performance, consistently achieving the highest R² values (averaging ~0.8) and the lowest error rates (Table 1). The high concordance between the predicted miRNA activity and the observed miRNA expression is visualized in Fig 1A. Furthermore, we assessed the model’s performance for each individual miRNA by calculating the Spearman correlation coefficient. The resulting distributions, shown in Fig 1B, reveal that the vast majority of miRNAs displayed a strong positive correlation (mean ρ > 0.5), confirming the fundamental ability of the XGBoost model to capture the complex regulatory relationships between mRNA and miRNA expression from bulk tissue data.

**Table 1:** Model comparison on different cancers.

Cancer Type	Model	MSE	MAE	R2
Breast Cancer	Ridge	0.028	0.115	0.709
	Lasso	0.027	0.114	0.718
	Random Forest	0.019	0.096	0.798
	Neural Network	0.022	0.105	0.771
	**XGBoost**	**0.012**	**0.062**	**0.821**
Lung Cancer	Ridge	0.026	0.116	0.712
	Lasso	0.043	0.158	0.523
	Random Forest	0.021	0.103	0.772
	Neural Network	0.023	0.112	0.742
	**XGBoost**	**0.011**	**0.080**	**0.821**
Ovarian Cancer	Ridge	0.024	0.106	0.775
	Lasso	0.029	0.130	0.730
	Random Forest	0.021	0.101	0.804
	Neural Network	0.023	0.109	0.785
	**XGBoost**	**0.020**	**0.096**	**0.814**
Prostate Cancer	Ridge	0.026	0.108	0.742
	Lasso	0.022	0.102	0.784
	Random Forest	0.020	0.096	0.801
	Neural Network	0.022	0.103	0.782
	**XGBoost**	**0.012**	**0.071**	**0.833**

A critical step in our study was to confirm that this predictive accuracy translates to the unique context of spatial transcriptomics. To this end, we performed an external validation using an independent breast cancer spatial transcriptomics dataset (STDS0000038) that, crucially, contains experimentally measured ground-truth miRNA expression. This allowed for a direct, quantitative comparison between STmiR’s predicted activity and actual measured miRNA levels for each spatial spot. Our analysis focused on four common miRNAs: hsa-mir-200c, hsa-mir-503, hsa-let-7b, and hsa-mir-210. As shown in S3 Fig, this direct validation yielded compelling results. We observed a strong and statistically significant positive Pearson correlation between the predicted miRNA activity and the true measured expression for all four miRNAs (R-values ranging from 0.559 to 0.708; p < 0.001 for all). Beyond quantitative correlation, we also assessed whether STmiR could recapitulate the spatial distribution of these miRNAs. A qualitative comparison of the UMAP plots revealed a high degree of similarity between the spatial patterns of predicted activity and true expression. This dual quantitative and qualitative evidence provides strong support for STmiR’s ability to accurately and reliably infer spatially resolved miRNA activity, establishing a solid foundation for its application in subsequent biological discovery.

### Pan-cancer analysis reveals conserved regulatory hubs and tissue-specific heterogeneity

**Fig 2:**
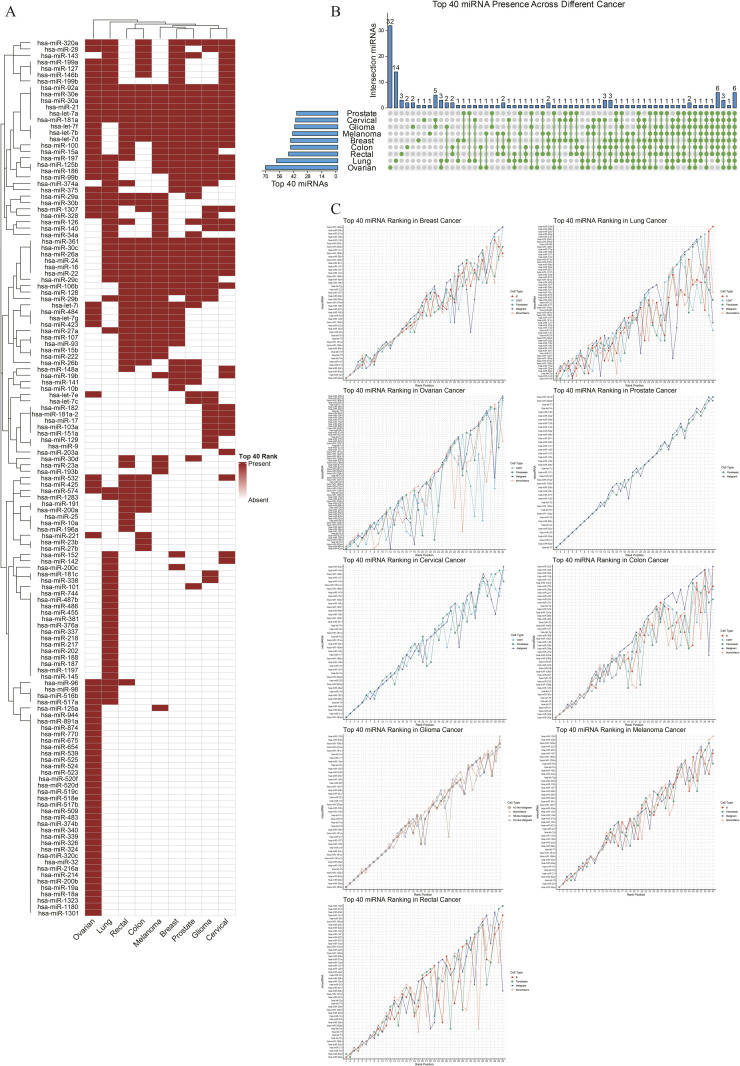
Pan-Cancer Spatial Comparison of miRNA Commonality and Heterogeneity. (A) Venn diagram illustrating six pan-cancer conserved miRNAs (e.g., hsa-miR-21, hsa-let-7a) consistently ranked in the top 40 across nine cancer types. (B) Heatmap displaying miRNA rankings across cancers. Red highlights conserved miRNAs (e.g., miR-30 family), while color gradients reflect cancer-specific variations. (C) Line plot showing average miRNA expression levels across cell types (fibroblasts, B cells, malignant cells) in nine cancers. Solid lines indicate conserved rankings in breast and cervical cancers; dashed lines highlight heterogeneity in lung and ovarian cancers.

Having established the model’s reliability, we applied STmiR to explore the landscape of miRNA regulation across nine distinct cancer types. This pan-cancer analysis aimed to identify miRNAs with conserved high activity, suggesting fundamental roles in tumorigenesis. By ranking the average predicted miRNA activity in each cancer type, we identified six miRNAs—hsa-miR-30a, hsa-miR-30e, hsa-miR-181a, hsa-let-7a, hsa-miR-92a, and the well-known onco-miRNA **hsa-miR-21**—that consistently ranked among the top 40 across all nine malignancies (Fig 2A, 2B). This remarkable conservation highlights their potential as core regulators in cancer biology. Indeed, these miRNAs represent key players in tumorigenesis; for instance, hsa-let-7a is a well-documented tumor suppressor that limits cell proliferation [30], whereas hsa-miR-21 is a potent oncogene that promotes tumor cell survival and growth [31]. Other identified miRNAs, including the miR-30 family, miR-181a, and miR-92a, are also widely implicated in modulating cell proliferation, differentiation, and migration within the tumor microenvironment [32–34]. The prominence of onco-miRNAs like hsa-miR-21 underscores the importance of dissecting these complex networks at the tissue level. To illustrate STmiR’s capability in this regard, we performed an in-depth case study of breast cancer, focusing on its most active miRNA regulatory networks.

Our pan-cancer analysis also highlighted the diversity of miRNA regulation. While some cancers, like breast and colon cancer, showed consistent miRNA activity rankings across their constituent cell types, others, such as lung and ovarian cancer, exhibited significant heterogeneity (Fig 2C). This suggests that in certain tumors, miRNA regulation is highly context-dependent, varying significantly among different cellular communities within the tumor microenvironment.

### Pan-cancer miRNA conservation in key cell subtypes

**Fig 3:**
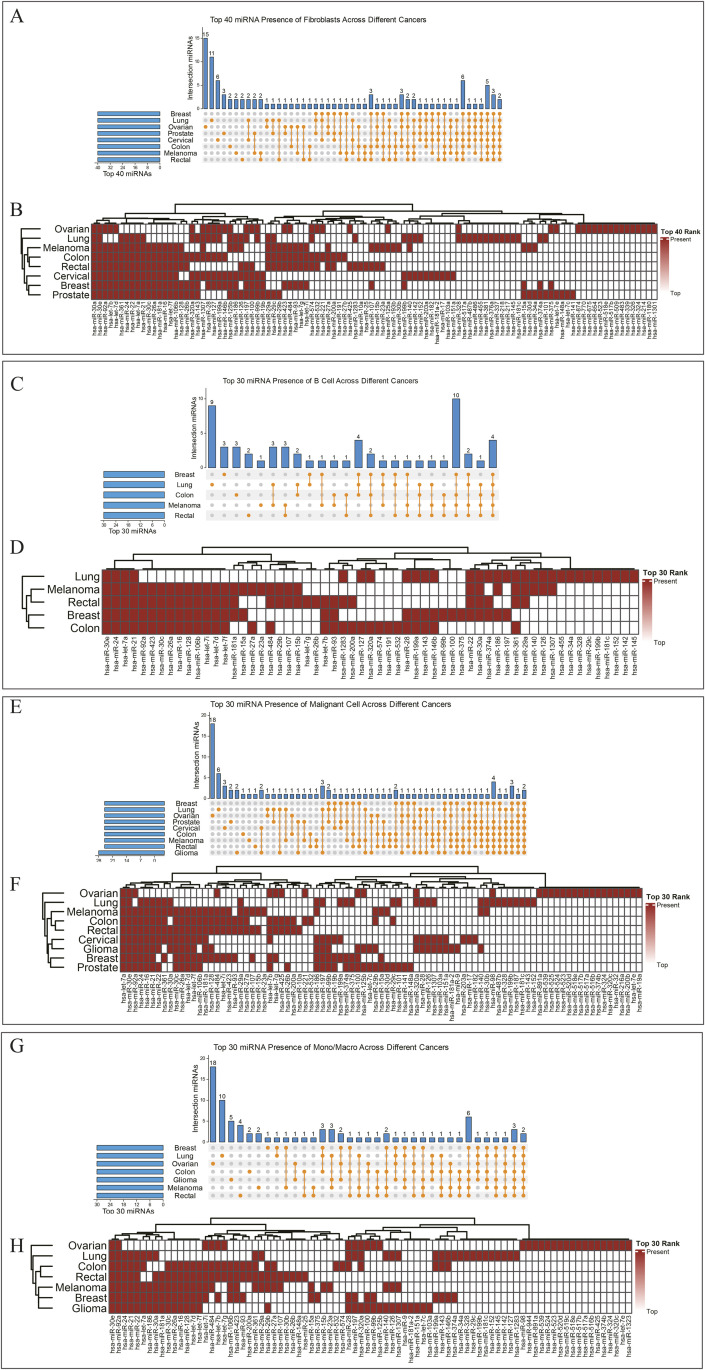
Pan-Cancer miRNA Conservation in Cell Subtypes. (A, B) Fibroblasts: Violin plots of hsa-miR-30a and hsa-miR-30e expression, conserved across cancers. These miRNAs regulate fibroblast activation and fibrosis. (C, D) B cells: Boxplots of hsa-let-7a (tumor suppressor) and hsa-miR-21 (oncogenic), showing balanced roles in immune modulation. (E, F) Malignant cells: Scatterplots of hsa-let-7a and hsa-miR-30e expression, linked to oncogene repression (e.g., RAS) and metastasis inhibition. (G, H) Monocytes/Macrophages: Heatmaps of miR-30e and miR-92a expression, indicating roles in macrophage polarization and immune response regulation.

To further dissect these patterns, we investigated conserved miRNA activity within specific cell subtypes across the different cancers. This analysis revealed consistent signatures in key cell populations of the tumor microenvironment (Fig 3).

- **In fibroblasts**, members of the miR-30 family, particularly hsa-miR-30a and hsa-miR-30e, were consistently active. This finding is significant as these miRNAs are known modulators of fibroblast activation and fibrosis, critical processes in shaping the supportive tumor stroma [35,36].
- **In B cells**, we observed a balance of activity between the tumor-suppressive hsa-let-7a and the oncogenic hsa-miR-21. This highlights the complex immunomodulatory role of B cells, where miRNAs simultaneously regulate both tumor-suppressive and pro-tumorigenic pathways within the immune compartment [37,38].
- **Within malignant cells** themselves, hsa-let-7a and hsa-miR-30e were the most consistently active miRNAs. The sustained activity of let-7a likely reflects a persistent, albeit often overcome, cellular mechanism to restrain oncogenes like RAS, while miR-30e has been implicated in regulating cell migration and invasion [39,40].
- **In monocytes/macrophages**, hsa-miR-30e and hsa-miR-92a showed conserved activity, implicating their roles in regulating macrophage polarization and the tumor’s immune landscape, potentially influencing the switch between anti-tumor M1 and pro-tumor M2 phenotypes [41–45].

### Cell-type-specific miRNA signatures highlight context-dependent regulation

**Fig 4:**
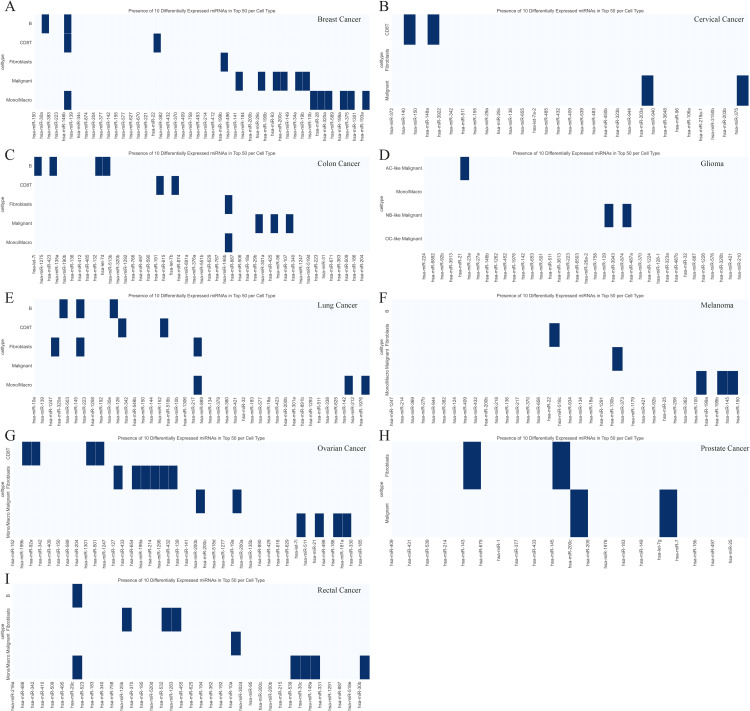
Cell-Type-Specific miRNA Signatures Across Cancers. (A) Breast cancer: hsa-miR-30a (B cells), hsa-miR-146b (B cells, CD8 + T cells, monocytes/macrophages), hsa-miR-22 (CD8 + T cells), and hsa-miR-199b (fibroblasts) delineate immune-stromal crosstalk. (B) Cervical **cancer**: CD8 + T cells express hsa-miR-140 and hsa-miR-148a (immune surveillance), while hsa-miR-203a (differentiation) and hsa-miR-375 (proliferation) dominate malignant cells. (C) Colon **cancer**: B cells exhibit hsa-let-7i, miR-423, and miR-132 (tumor suppression), with hsa-miR-146b shared across fibroblasts and monocytes/macrophages (inflammation/remodeling). (D) **Glioma**: Subtype-specific signatures include hsa-miR-21 (AC-like) and hsa-miR-129 (NB-like), indicating divergent oncogenic pathways. (E) Lung cancer: hsa-miR-320a/hsa-miR-145 (B cells), hsa-miR-217 (fibroblast activation), and hsa-miR-200c (malignant cells, EMT) highlight microenvironmental interplay. (F) Melanoma: hsa-miR-22 (fibroblasts) and hsa-miR-130b (malignant cells, metastasis) underscore stromal-tumor dynamics. (G) Ovarian cancer: Fibroblast-enriched miRNAs (hsa-miR-127, hsa-miR-654, hsa-miR-199a) and hsa-miR-21 (dual stromal/immune roles) reflect TME complexity. (H) Prostate **cancer**: hsa-miR-143/hsa-miR-145 mark fibroblasts, linked to desmoplastic stroma. (I) Rectal **cancer**: hsa-miR-125b/hsa-miR-532 (fibroblasts), hsa-miR-10a (malignant cells), and hsa-miR-539 (monocytes/macrophages) implicate inflammation-driven TME remodeling.

Beyond the conserved patterns, our analysis also identified unique miRNA activity signatures specific to certain cell types within individual cancers, underscoring the context-dependent nature of miRNA regulation (Fig 4). For instance, in lung cancer, hsa-miR-200c activity was a specific marker for malignant cells, likely related to its well-established role in suppressing the epithelial-mesenchymal transition (EMT), a key process in metastasis [46]. In contrast, in prostate cancer, hsa-miR-143 and hsa-miR-145 were highly active specifically in fibroblasts, reflecting the desmoplastic stromal reaction characteristic of that disease. These results showcase STmiR’s capacity to uncover nuanced, tissue-specific regulatory programs that would be obscured in bulk analysis.

### Case study: Dissecting miRNA regulatory networks in breast cancer

To demonstrate STmiR’s utility in generating specific, testable hypotheses, we performed an in-depth case study in breast cancer. We analyzed the predicted activity of the most prominent miRNAs within four major cell types: malignant cells, B cells, fibroblasts, and myofibroblasts.

**Fig 5:**
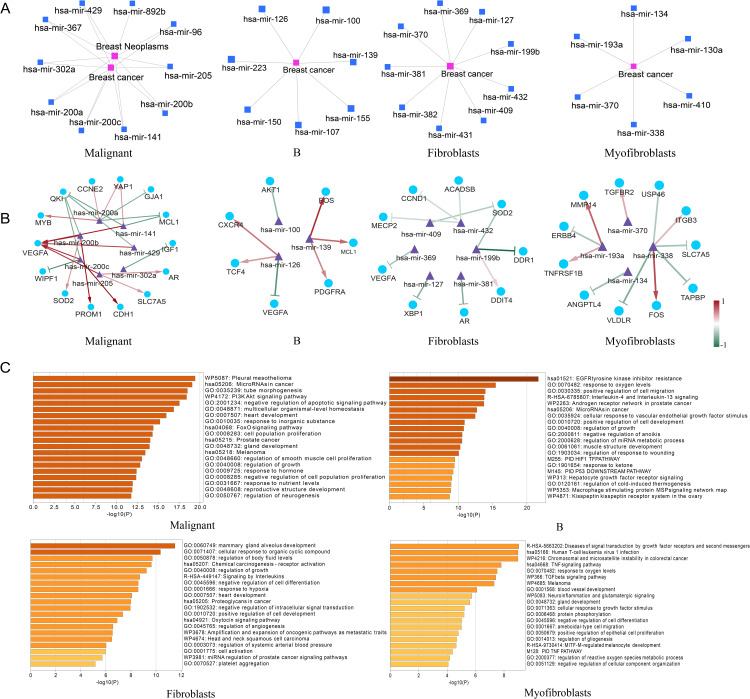
Breast cancer top miRNAs-target gene and miRNAs-disease regulatory network. (A) Network diagram linking top differentially expressed miRNAs (malignant cells, B cells, fibroblasts, myofibroblasts) to breast cancer pathways. (B) miRNA-target interaction map: Red edges (positive Pearson correlation, e.g., miR-205-AR), green edges (negative correlation, e.g., miR-200b-LOX). Line darkness reflects correlation strength. (C) Pathway enrichment bar charts: Malignant cells enrich “PI3K-Akt signaling,” B cells associate with “EGFR resistance,” fibroblasts link to “mammary gland development,” and myofibroblasts to “TNF signaling.”.

Our analysis of the malignant cell population revealed a complex regulatory network (Fig 5A**, left panel**) where members of the oncogenic miR-200 family (miR-200a/b/c) and miR-141 showed high predicted activity. Notably, our network recapitulated several experimentally validated interactions from existing literature. For instance, the predicted negative regulation of the EMT-associated gene CDH1 by the miR-200 family is a cornerstone of cancer metastasis research [47]. Similarly, our identified link between miR-205 and the Androgen Receptor (AR) aligns with studies suggesting their expression levels could serve as predictive markers in breast cancer [48]. Furthermore, the predicted targeting of YAP1 by miR-200a is consistent with reports showing their inverse correlation in clinical specimens [49]. To understand the functional implications of these networks, we performed pathway enrichment analysis on the predicted target genes (Fig 5B). For malignant cells, the targets were significantly enriched in critical cancer pathways, including “MicroRNAs in cancer,” “PI3K-Akt signaling pathway,” and “FoxO signaling pathway,” confirming that our predictions capture biologically coherent regulatory modules.

Similarly, we identified distinct miRNA networks and associated pathways for B cells, fibroblasts, and myofibroblasts (Fig 5), highlighting how different cell types within the TME are governed by unique regulatory logic. For example, the predicted targeting of VEGFA by miR-126 in B-cells is consistent with its known role in angiogenesis [50]. This case study demonstrates how STmiR can move beyond single-molecule analysis to deconstruct the complex, cell-type-specific miRNA-mediated regulatory networks that collectively drive tumor biology, thereby generating rich, spatially-resolved hypotheses for future experimental validation.

## Discussion

In this study, we developed and validated STmiR, a novel machine learning framework based on XGBoost, to infer miRNA activity from spatial transcriptomics data. By successfully bridging the gap left by technologies not optimized for direct miRNA capture, STmiR provides a powerful approach to explore the spatial dimension of post-transcriptional regulation within the native tissue context. Our work not only demonstrates the technical feasibility and accuracy of this prediction—validated against both bulk data and, crucially, ground-truth spatially resolved miRNA measurements—but also applies it to uncover biologically meaningful patterns across a pan-cancer atlas.

Our pan-cancer analysis revealed a core set of miRNAs, including the well-documented onco-miRNA hsa-miR-21 and the tumor-suppressive let-7 family, that exhibit conserved high activity across nine distinct malignancies. The consistent prominence of these miRNAs suggests their involvement in fundamental oncogenic processes that are shared across different tumor types. The dynamic balance between oncogenic miRNAs like miR-21, which drives proliferation and inhibits apoptosis [31], and tumor-suppressive miRNAs like the let-7 family, which restrain key oncogenes like RAS [39], likely represents a central regulatory battlefield in tumorigenesis. Our ability to spatially map the activity of these opposing forces provides a new lens through which to view tumor progression. Furthermore, the observed heterogeneity in miRNA activity landscapes, particularly in lung and ovarian cancers, underscores the profound impact of the tumor microenvironment (TME) [51,52]. This suggests that while core oncogenic pathways may be conserved, their regulation by miRNAs can be highly plastic and dependent on the unique cellular composition and crosstalk within a specific TME, a notion supported by recent studies on spatial intratumor heterogeneity.

A key strength of STmiR is its ability to deconvolve miRNA activity within specific cell types, moving beyond the bulk-level view. Our analyses consistently highlighted distinct miRNA regulatory programs operating in malignant cells versus various stromal and immune components of the TME. For example, the high activity of the miR-30 family in fibroblasts across multiple cancers points to a conserved role in regulating stromal responses and extracellular matrix remodeling, potentially driving the desmoplastic reaction seen in many solid tumors [35]. Similarly, the specific miRNA signatures identified in B cells and macrophages highlight the intricate role of miRNA-mediated regulation in shaping tumor immunology [45]. The breast cancer case study further solidified this utility. By dissecting the complex regulatory networks within four distinct cell populations, we moved from a single-molecule view to a systems-level understanding. We demonstrated how STmiR can generate highly specific, data-driven hypotheses—such as the interplay between the miR-200 family in malignant cells and pathways like PI3K-Akt signaling—that are primed for subsequent experimental validation.

Methodologically, STmiR offers a robust and computationally efficient alternative to other advanced techniques. While deep learning models may offer advantages in capturing highly complex, non-linear patterns or integrating multi-modal data like histology images [53, 54], our XGBoost-based approach provides a balance of high accuracy, interpretability, and robustness to the inherent sparsity and noise of ST data. The successful validation against measured spatial miRNA data confirms that a model trained on large-scale bulk datasets can indeed learn generalizable regulatory principles that are transferable to the spot-level microenvironment. This aligns with a growing trend of using computational methods to deconvolve complex tissue architecture and molecular interactions from spatial data [55,56] and positions STmiR as a valuable and accessible tool for researchers to conduct large-scale exploratory analyses of miRNA function. The potential clinical relevance of identifying such spatially-defined regulatory hubs is significant [57].

### Limitations of the study

Despite the promising results, we acknowledge several limitations inherent to our study. A primary limitation is the domain shift between the bulk RNA-seq data used for training and the spot-level data used for prediction. We must critically consider the potential pitfalls of this approach. Bulk tissue samples represent an averaged expression profile from millions of cells, whereas an ST spot, while not single-cell, captures a unique microenvironment with specific cellular compositions and interactions. This difference in cellular heterogeneity and technical noise profiles could potentially introduce biases into the predictions.

We have sought to mitigate this issue through several strategies. The XGBoost algorithm is inherently robust to noise and data sparsity. More importantly, our key biological conclusions are not derived from single-spot predictions but from statistical trends and correlations across thousands of spots. Our “predict-then-associate” method for inferring cell-type-specific activity is a conservative approach that avoids applying the bulk-trained model directly to deconvoluted profiles. However, the gold standard for validation remains direct experimental measurement. While our computational validation against the STDS0000038 dataset provides strong support, future work should aim to validate specific findings—such as the cell-type-specific activity of hsa-miR-21 in breast cancer—using techniques like multiplexed *in situ* hybridization (e.g., RNAScope) or immunofluorescence co-localization for target proteins, as has been demonstrated as feasible in recent spatial multi-omics studies [14].

Furthermore, our cell-type attribution method, which links miRNA activity to the dominant cell type in a spot, is a conservative simplification. In spots with highly mixed populations, this approach may overlook signals from sub-dominant but biologically important cell types. Future methods could aim to deconvolve activity in a more proportional manner.

## Conclusion

In conclusion, STmiR represents a significant step forward in our ability to understand the spatial regulation of miRNAs in cancer. By accurately inferring miRNA activity in a spatially resolved manner, this approach provides a powerful resource for exploring the intricate regulatory networks that govern gene expression in complex tissues. The work presented here not only offers novel biological insights into pan-cancer miRNA regulation but also lays a robust methodological foundation for future research. By generating rich, spatially-contextualized, and testable hypotheses, STmiR opens new avenues for investigating the roles of miRNAs in tumor progression, microenvironment remodeling, and therapeutic response.

**Resources and methods**

## Supporting information

S1 Supporting information. (DOCX)

S1 Fig The overall workflow of the STmiR framework. This Fig illustrates the three main stages of the STmiR methodology. The process begins with model construction, where paired miRNA-mRNA bulk RNA-seq data from TCGA and CCLE are integrated to train an XGBoost model that uses mRNA expression to predict miRNA activity. Next, in the spatial transcriptomics application stage, this pre-trained model is used to infer miRNA activity from the mRNA expression profiles of spatial spots, while cell-type deconvolution is concurrently performed using cell2location. In the final stage, the framework enables downstream functional analysis by identifying cell-type-specific miRNA activity to construct miRNA-target gene and miRNA-disease regulatory networks for biological interpretation.
(PDF)

S2 Fig Schematic of the model construction and evaluation pipeline. This diagram outlines the procedure for building and validating the predictive model. The workflow starts with paired miRNA-mRNA profiles derived from bulk RNA-seq samples. The mRNA expression data serves as the input for model construction, which generates a predicted miRNA profile. This predicted profile is then compared against the actual miRNA profile from the same samples, which serves as the ground truth. The predictive performance of the model is quantitatively assessed by calculating the Spearman correlation between the predicted and actual miRNA profiles.
(PDF)

S3 Fig Direct Validation of STmiR’s Predictive Accuracy on Spatial miRNA Data. The Fig demonstrates the model’s performance by comparing predicted miRNA expression to true measured expression from an independent spatial transcriptomics dataset. (A-D) Scatterplots show the correlation between predicted (y-axis) and true (x-axis) scaled expression for four miRNAs: (A) hsa-mir-200c (R = 0.708, p = 0.000), (B) hsa-mir-503 (R = 0.608, p = 0.000), (C) hsa-let-7b (R = 0.642, p = 0.000), and (D) hsa-mir-210 (R = 0.559, p = 0.000). The red line indicates the linear regression fit. (E-H) UMAP plots illustrate the spatial distribution of the true measured expression for (E) hsa-mir-200c, (F) hsa-mir-503, (G) hsa-let-7b, and (H) hsa-mir-210. (I-L) UMAP plots illustrate the spatial distribution of the STmiR-predicted expression for (I) hsa-mir-200c, (J) hsa-mir-503, (K) hsa-let-7b, and (L) hsa-mir-210. The similarity in spatial patterns between the true (E-H) and predicted (I-L) plots confirms the model’s ability to capture spatial localization of miRNA activity.
(PDF)

S4 Raw data. (ZIP)

S5 Supporting information. (DOCX)
