# Supplementary material for: STmiR: A Novel XGBoost-based framework for spatially resolved miRNA activity prediction in cancer transcriptomics
Source: PLoS One. 2025 Sep 9;20(9):e0322082. doi: 10.1371/journal.pone.0322082 (PMC12419590; doi:10.1371/journal.pone.0322082)
Supplement: S1 — (DOCX) [file pone.0322082.s001.docx]

### Supplement Materials

### Method details

**1.Workflow of STmiR**

The architecture of STmiR is illustrated in **Figure S1**. STmiR is a universal computational framework designed to infer spatially resolved miRNA expression using bulk RNA-seq, scRNA-seq, and spatial transcriptomics data. Detailed methods are provided in the Resources and Methods section. STmiR begins with preprocessing paired miRNA and mRNA profiles for each cancer type. Then, it constructs a predictive model for each cancer type to infer miRNA activity based on bulk RNA-seq data. Once the model is trained, it is transferred to spatial transcriptomics data to predict miRNA activity. To enhance the interpretability of our STmiR framework, we incorporate the cell2location algorithm to annotate cell types within the spatial transcriptomics data. STmiR's precision is validated on independent cancer datasets, focusing on the correlation between predicted miRNA expression and observed target-mRNA expression. A comprehensive regulatory network is also constructed to delineate miRNAs' roles in cancer, detailing interactions between miRNAs and their targets within cancer cells and extending to miRNA-disease associations.

**2.****Data collection**

**Bulk RNA-seq and miRNA-seq**

We obtain matched bulk RNA-Seq and miRNA-Seq gene expression data for cell lines (Cancer Cell Line Encyclopedia, CCLE (Barretina et al., 2012)) and Pan-cancer (TCGA) from URLs [https://pancanatlas.xenahubs.net](https://pancanatlas.xenahubs.net/). Batch effects normalized mRNA-seq data and miRNA-seq data are obtained from TCGA. Among them, miRNA sequencing data contain 744 miRNAs across 10818 samples, and mRNA sequencing data contain 20532 genes across 11060 samples. TPM (Transcripts Per Kilobase Million) normalized RNA-seq data is utilized from CCLE(Ghandi et al., 2019). Firstly, after outlier processing and logarithmic transformation of the original TCGA and CCLE data, we select matched mRNA and miRNA samples from four different sites: breast cancer, lung cancer, ovarian cancer, and prostate cancer. Subsequently, considering the miRNA names, we remove the long suffixes (e.g., -3p), then remove duplicates of the same miRNA name and replace them with the median value. Finally, we employ rank-based methods to integrate the sequencing data from both TCGA and CCLE sources, aiming to eliminate batch effects. Our study is firmly anchored in the extensive repository miRbase (Kozomara et al., 2019) of miRNAs, which serves as a cornerstone for the annotation of miRNAs in our study. To mitigate the occurrence of false positives within the miRbase database, we have harnessed the principle of evolutionary conservation of miRNAs, a concept articulated by (Bartel, 2004). This biological criterion is instrumental in distinguishing true miRNA candidates from potential artifacts.

**scRNA-seq**

We utilize four individual single-cell gene sequencing expression profiles from the TISCH2 database (<http://tisch.comp-genomics.org/gallery/>). It provides differential gene expression, cell-type annotation, and meta information. Pointedly, gene expressions for breast cancer (ID=T020088) and ovarian cancer (ID=T020149) are obtained from (Zhang et al., 2014) study. The single-cell mRNA data for non-small cell lung cancer(ID=T010056) are sourced from the study by (Lambrechts et al., 2018). Additionally, single-cell RNA-Seq data with cell type annotation for prostate cancer (ID=T020163) are obtained from the study by (Dong et al., 2020). Especially， the scRNA-seq data of the four cancers we download must contain malignant cells. To convert single-cell RNA sequencing expression data from h5 files to loom files, we use the Seurat package and the loomR package and then perform subsequent analysis and processing.

**Spatial transcriptomics**

10X Visium Spatial Transcriptomics (ST) data are downloaded from the 10x genomics([https://www.10xgenomics.com/datasets](mailto:https://www.10xgenomics.com/datasets)). We select the ST expression data of breast cancer, non-small cell lung cancer, ovarian cancer, and prostate cancer. For each cancer, we download the spatial imaging data and the filter feature matrix file. The gene-spot matrices generated after ST data processing from ST and Visium samples are analyzed with the Scanpy(Wolf et al., 2018) package. We perform some basic filtering of spots based on total counts and expressed genes.

Among the reads of the breast cancer spatial transcriptome sequencing data, we identify some outliers. therefore, we control the read counts to reside within a predefined range. Specifically, in processing ST expression data for breast cancer, we filter out five cells with expression counts exceeding 38,000. Furthermore, we exclude 2,988 genes expressed in fewer than ten cells, ensuring that only the most robust and consistently expressed genes are considered in our analysis. Then, we normalize the Visium count data using the standardization method built into the Scanpy package and perform a log10 transformation. This transformation helps reduce the dynamic range of the data, making subsequent analyses more stable and sensitive. To identify the most discriminative features, we identify highly variable genes and select the top 2,000 feature genes. These genes exhibit significant expression differences between different cells, which are crucial for subsequent cell type identification and biological process analysis. Finally, we scale the expression data. In our subsequent analysis, we conduct Uniform Manifold Approximation and Projection (UMAP) to reduce dimensionality based on gene expression information, followed by clustering.

**3.Model construction and performance evaluation**

We choose to utilize Extreme Gradient Boost (XGBoost) due to its ability to cope with sparse data, mitigate overfitting, and provide faster training (T. Chen & Guestrin, 2016). XGBoost is an ensemble machine learning model based on decision trees, it consists of a series of decision trees and combines them to form a robust prediction model. Within the XGBoost algorithm, it iteratively builds a new decision tree according to loss for prediction and ground truth established by the preceding model(T. Chen & Guestrin, 2016).

In our study, we employ the XGBoost package in Python to construct our predictive model, using bulk RNA-seq data comprising paired mRNA and miRNA expression profiles. The mRNA expression data are utilized as input features for the XGBoost model, with miRNA activity is used as the output variable. We implement the Grid Search method to exhaustively identify the optimal hyperparameter combination for the model, as detailed by (Putatunda & Rama, 2018). One important point to note is that we integrate bulk RNA-seq data from TCGA and CCLE, focusing on genes that overlap between them. Before integration, we apply rank-based operations to each dataset to mitigate batch effects.

To enhance the precision of miRNA annotations and the biological relevance of our findings, we curate the miRNA database by selecting miRNAs present in both TCGA and CCLE datasets. This approach not only minimizes the potential for false positive annotations, as highlighted by (Fromm et al., 2022), but also ensures a comprehensive representation of miRNAs biologically significant to the cancer types studied. Moreover, the mRNA features we select intersect with spatial transcriptomics data. Initially, we apply min-max standardization to the paired bulk mRNA and miRNA sequencing data, followed by the division of the dataset into training and testing sets. During model development, we use default parameters and determine additional hyperparameters via grid search. Post-training, we calculate the Spearman correlation coefficient between the predicted miRNA activity and actual values (**Figure S2**).

Furthermore, we develop comparative models, including ridge regression, Lasso regression, random forest regression, and neural network regression, to benchmark against the XGBoost model. The performance of these models is assessed by calculating the Mean Squared Error (MSE), Mean Absolute Error (MAE), and the coefficient of determination (R²). Additionally, we visualize the XGBoost model's fitting curve to evaluate its predictive accuracy.

**4.Application of the model to spatial transcriptomics**

To apply a bulk-trained model to ST data, we first select the overlapped genes between bulk RNA-seq and spatial transcriptomics data. After completing the model training, we feed the spatial transcriptomics expression data into the pre-trained XGBoost model to obtain predicted miRNA activity.

Additionally, we plan to perform cell type annotation on spatial transcriptomics data to obtain abundance information for different cell types in each spot, thus exploring the impact of different cell types on cancer miRNA. For this purpose, we have chosen the cell2location algorithm as our tool. Cell2location is a Bayesian-based model. It not only captures the biological variability among different cell types but also incorporates the technical effects that are inherent in the generation of spatial transcriptomics data, as articulated by (Kleshchevnikov et al., 2022).

We conducted a comprehensive differential expression analysis on a range of malignant and non-malignant cell types, including cancer cells, fibroblasts, myofibroblasts, and B cells, for each cancer type. This analysis enabled us to identify miRNAs that were significantly differentially expressed. Subsequently, we mapped a regulatory network of miRNAs and diseases using the miRNet platform (<https://www.mirnet.ca/>). To elucidate the functions of these miRNAs, we utilized the HMDD database (Cui et al., 2024) to extract target genes associated with each miRNA. We further performed a pathway enrichment analysis for these target genes with Metascape (Zhou et al., 2019). Ultimately, by assessing the correlation between the predicted miRNA activity and the expression levels of their target genes, we deduced potential regulatory relationships. A positive correlation suggests that the miRNA may promote the expression of its target gene, whereas a negative correlation implies that the miRNA may suppress or dysregulate its target gene expression.
