# Supplementary material for: STmiR: A Novel XGBoost-based framework for spatially resolved miRNA activity prediction in cancer transcriptomics
Source: PLoS One. 2025 Sep 9;20(9):e0322082. doi: 10.1371/journal.pone.0322082 (PMC12419590; doi:10.1371/journal.pone.0322082)
Supplement: S1 Fig — This Fig illustrates the three main stages of the STmiR methodology. The process begins with model construction, where paired miRNA-mRNA bulk RNA-seq data from TCGA and CCLE are integrated to train an XGBoost model that uses mRNA expression to predict miRNA activity. Next, in the spatial transcriptomics application stage, this pre-trained model is used to infer miRNA activity from the mRNA expression profiles of spatial spots, while cell-type deconvolution is concurrently performed using cell2location. In the final stage, the framework enables downstream functional analysis by identifying cell-type-specific miRNA activity to construct miRNA-target gene and miRNA-disease regulatory networks for biological interpretation. (PDF) [file pone.0322082.s002.pdf]

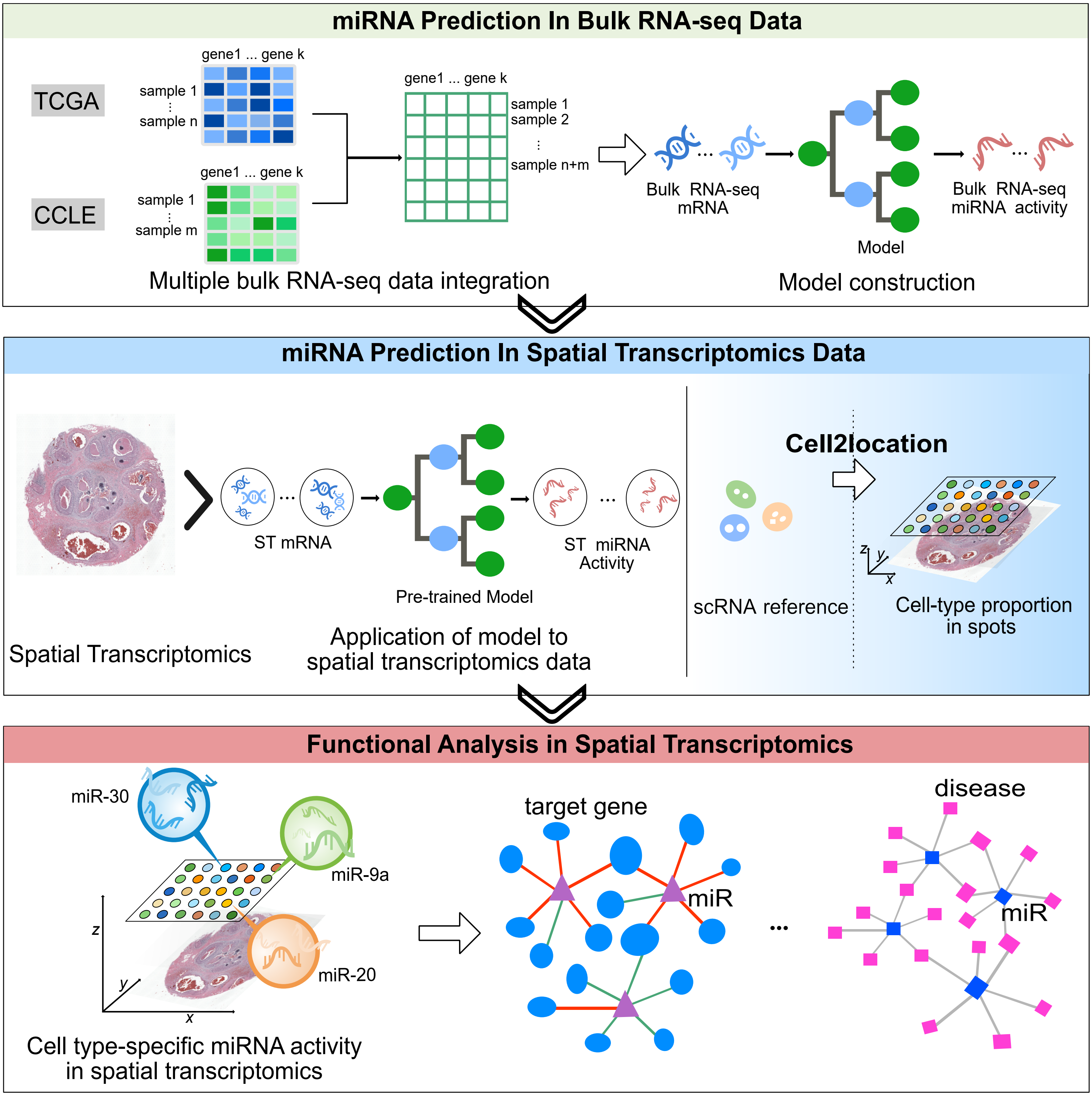

miRNA Prediction In Bulk RNA-seq Data
TCGA
CCLE
gene1 ... gene k
sample 1
sample n
sample m
sample 2
sample n+m
Bulk RNA-seq mRNA
Model
Bulk RNA-seq miRNA activity
Multiple bulk RNA-seq data integration
Model construction
miRNA Prediction In Spatial Transcriptomics Data
ST mRNA
ST miRNA Activity
Pre-trained Model
Spatial Transcriptomics
Application of model to spatial transcriptomics data
Cell2location
scRNA reference
Cell-type proportion in spots
Functional Analysis in Spatial Transcriptomics
miR-30
miR-9a
miR-20
Cell type-specific miRNA activity in spatial transcriptomics
target gene
miR
disease
