# Supplementary figures and images for: STmiR: A Novel XGBoost-based framework for spatially resolved miRNA activity prediction in cancer transcriptomics
Source: PLoS One. 2025 Sep 9;20(9):e0322082. doi: 10.1371/journal.pone.0322082 (PMC12419590; doi:10.1371/journal.pone.0322082)

## Bulk RNA-seq

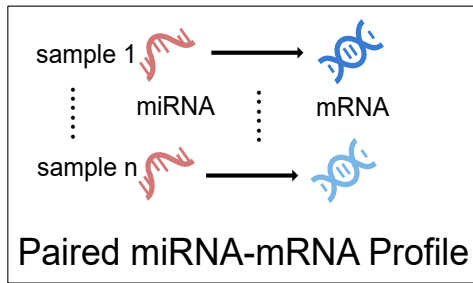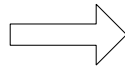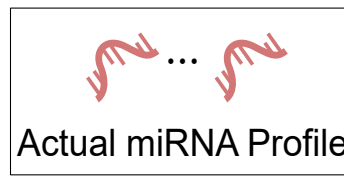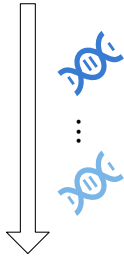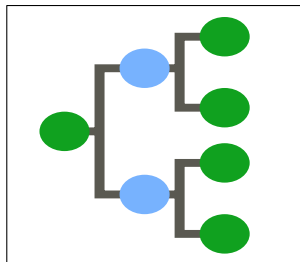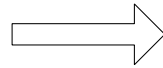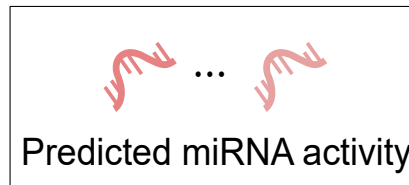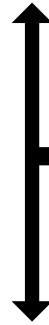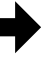

## Spearman Correlation

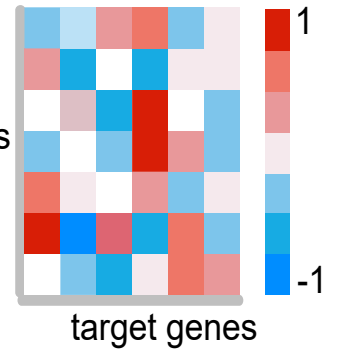

Supplement: S2 Fig — This diagram outlines the procedure for building and validating the predictive model. The workflow starts with paired miRNA-mRNA profiles derived from bulk RNA-seq samples. The mRNA expression data serves as the input for model construction, which generates a predicted miRNA profile. This predicted profile is then compared against the actual miRNA profile from the same samples, which serves as the ground truth. The predictive performance of the model is quantitatively assessed by calculating the Spearman correlation between the predicted and actual miRNA profiles. (PDF) [file pone.0322082.s003.pdf]

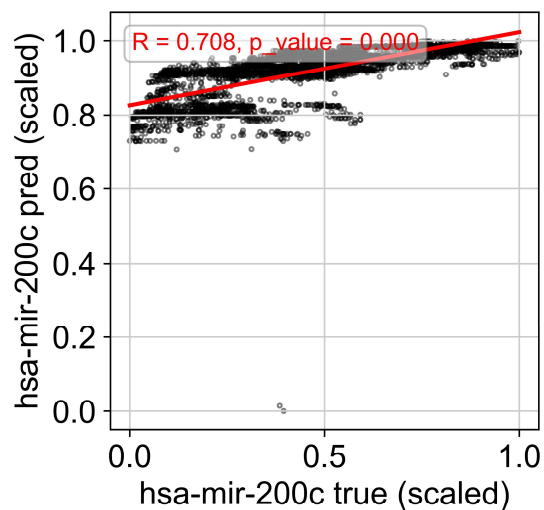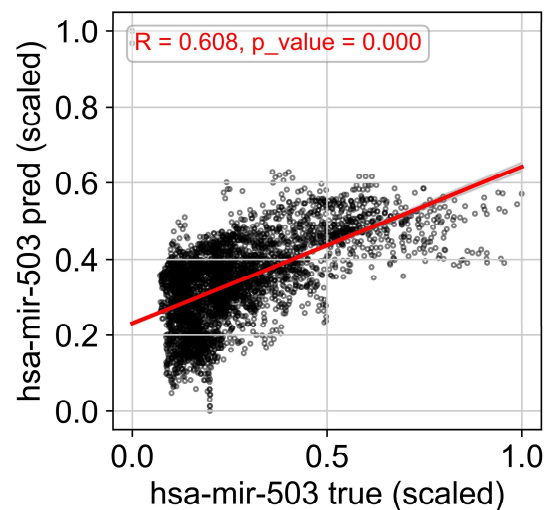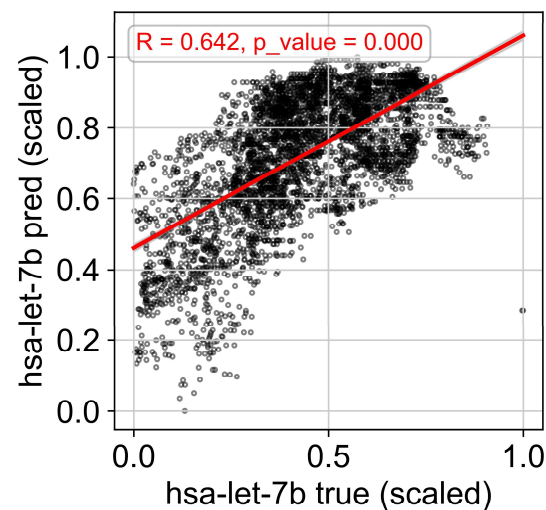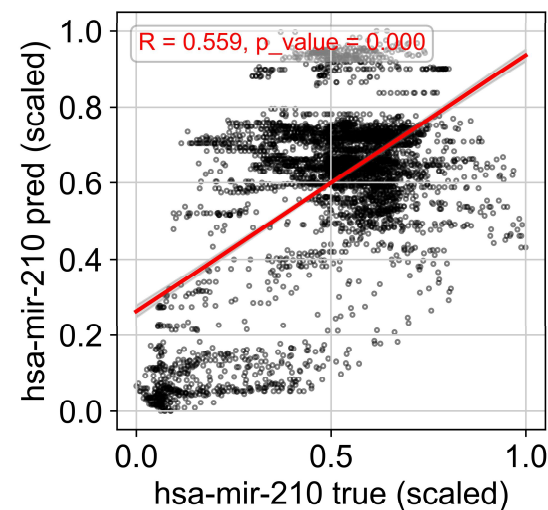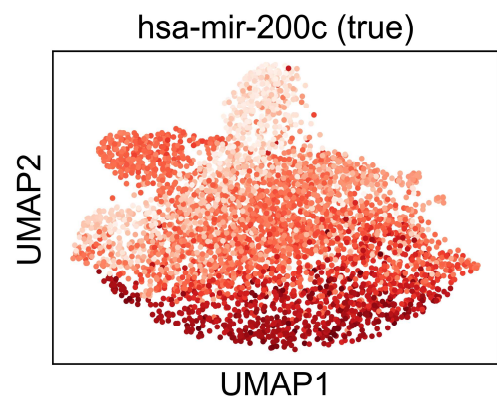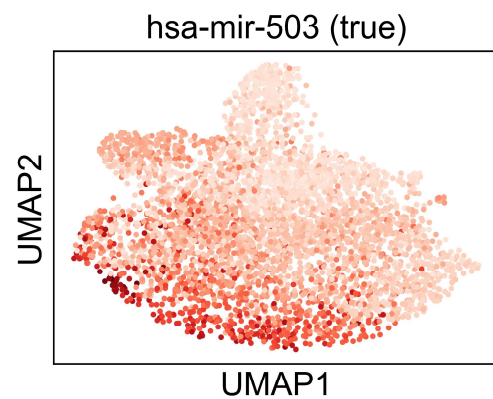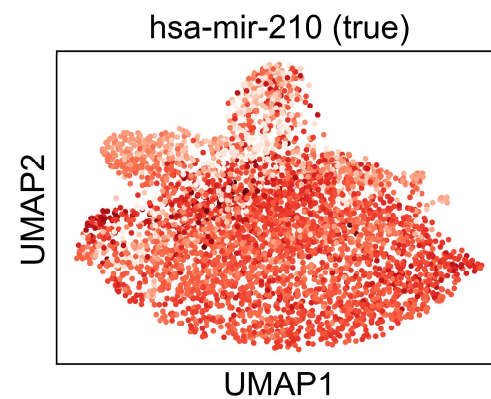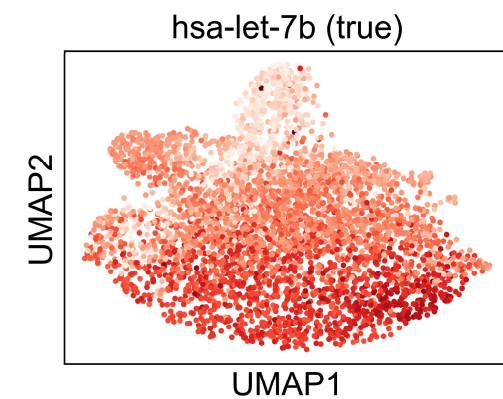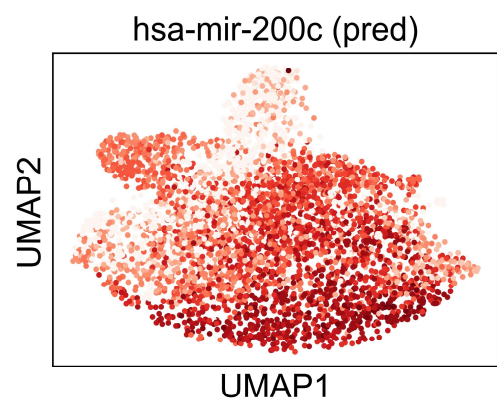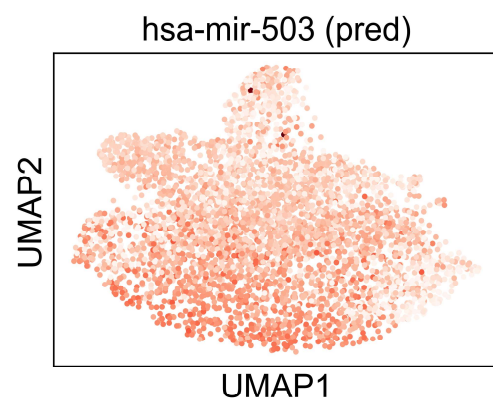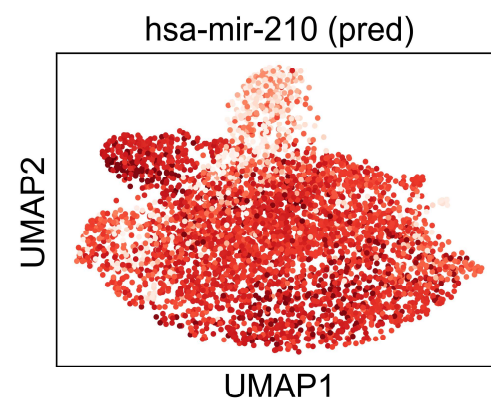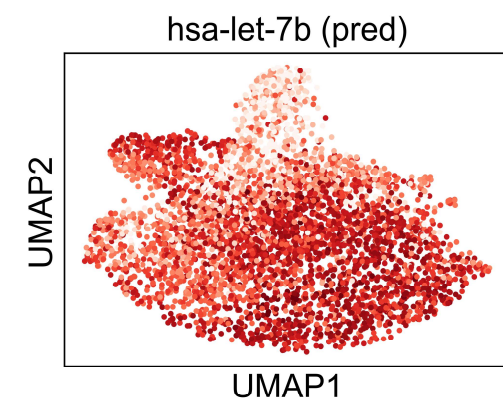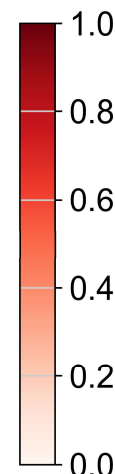

Supplement: S3 Fig — The Fig demonstrates the model’s performance by comparing predicted miRNA expression to true measured expression from an independent spatial transcriptomics dataset. (A-D) Scatterplots show the correlation between predicted (y-axis) and true (x-axis) scaled expression for four miRNAs: (A) hsa-mir-200c (R = 0.708, p = 0.000), (B) hsa-mir-503 (R = 0.608, p = 0.000), (C) hsa-let-7b (R = 0.642, p = 0.000), and (D) hsa-mir-210 (R = 0.559, p = 0.000). The red line indicates the linear regression fit. (E-H) UMAP plots illustrate the spatial distribution of the true measured expression for (E) hsa-mir-200c, (F) hsa-mir-503, (G) hsa-let-7b, and (H) hsa-mir-210. (I-L) UMAP plots illustrate the spatial distribution of the STmiR-predicted expression for (I) hsa-mir-200c, (J) hsa-mir-503, (K) hsa-let-7b, and (L) hsa-mir-210. The similarity in spatial patterns between the true (E-H) and predicted (I-L) plots confirms the model’s ability to capture spatial localization of miRNA activity. (PDF) [file pone.0322082.s004.pdf]
