# Supplementary material for: STmiR: A Novel XGBoost-based framework for spatially resolved miRNA activity prediction in cancer transcriptomics
Source: PLoS One. 2025 Sep 9;20(9):e0322082. doi: 10.1371/journal.pone.0322082 (PMC12419590; doi:10.1371/journal.pone.0322082)
Supplement: S5 — (DOCX) [file pone.0322082.s006.docx]

**SUPPLEMENTAL INFORMATION**

| RESOURCE | SOURCE | IDENTIFIER |  |  |
| --- | --- | --- | --- | --- |
| Deposited data | | |  |  |
| Cancer bulk RNA-seq | TCGA | [https://pancanatlas.xenahubs.net](https://pancanatlas.xenahubs.net/) |  |  |
| Cancer bulk miRNA-seq | TCGA | [https://pancanatlas.xenahubs.net](https://pancanatlas.xenahubs.net/) |  |  |
| Cell line bulk RNA-Seq | CCLE | <https://depmap.org/portal/download/all> |  |  |
| Cell line bulk miRNA-Seq | CCLE | <https://depmap.org/portal/download/all> |  |  |
| Breast Carcinoma scRNA-seq | TISCH2 | <http://tisch.comp-genomics.org/gallery/> |  |  |
| Non-small Cell Lung Cancer scRNA-seq | TISCH2 | <http://tisch.comp-genomics.org/gallery/> |  |  |
| Ovarian Serious Cystadenocarcinoma scRNA-seq | TISCH2 | <http://tisch.comp-genomics.org/gallery/> |  |  |
| Prostate Adenocarcinoma scRNA-seq | TISCH2 | <http://tisch.comp-genomics.org/gallery/> |  |  |
| Human Lung Cancer Spatial Gene Expression | 10x Genomics | <https://www.10xgenomics.com/datasets> |  |  |
| Human Ovarian Cancer Spatial Gene Expression | 10x Genomics | <https://www.10xgenomics.com/datasets> |  |  |
| Human Prostate Cancer Spatial Gene Expression | 10x Genomics | <https://www.10xgenomics.com/datasets> |  |  |
| Human Breast Cancer Spatial Gene Expression | 10x Genomics | <https://www.10xgenomics.com/datasets> |  |  |
| miRNA-disease correction | HMDD v4.0 | <https://www.cuilab.cn/hmdd> |  |  |
| Software and algorithms | | |  | Software and algorithms |
| STmiR | This paper | <https://github.com/Zero-plus-Seven/STmiR> |  |  |
| XGBoost | Conda | <https://github.com/dmlc/xgboost/> |  |  |
| Seurat | CRAN | [https://cran.r-project.org/web/ packages/Seurat/index.html](https://cran.r-project.org/web/%20packages/Seurat/index.html) |  |  |
| miRbaseConverter | Bioconductor | [https://www.bioconductor.org/packages/ release/bioc/html/miRBaseConverter.html](https://www.bioconductor.org/packages/%20release/bioc/html/miRBaseConverter.html) |  |  |
| ggplot | CRAN | [https://cran.r-project.org/web/ packages/ggplot2/index.html](https://cran.r-project.org/web/%20packages/ggplot2/index.html) |  |  |
| scanpy | Pypi | <https://pypi.org/project/scanpy/> |  |  |
| cell2location | Pypi | <https://pypi.org/project/cell2location/> |  |  |
| tydiverse | CRAN | <https://cran.r-project.org/web/packages/tidyverse/index.html> |  |  |
| BiocManager | CRAN | <https://cran.r-project.org/web/packages/BiocManager/index.html> |  |  |
| clusterProfiler | CRAN | [https://bioconductor.org/packages/ release/bioc/html/clusterProfiler.html](https://bioconductor.org/packages/%20release/bioc/html/clusterProfiler.html) |  |  |
| loomR | CRAN | [https://github.com/mojaveazure/loomR](mailto:https://github.com/mojaveazure/loomR) |  |  |
| Cytoscape | cytoscape | <https://cytoscape.org/download.html> |  |  |
| Metascape | metascape | <https://metascape.org/gp/index.html#/main/step1> |  |  |
| miRNet | mirnet | <https://www.mirnet.ca/> |  |  |
